# Breaking the error chain with SEE: cascade analysis of endodontic errors in clinical training

**DOI:** 10.1080/10872981.2023.2268348

**Published:** 2023-10-08

**Authors:** Abubaker Qutieshat, Gurdeep Singh

**Affiliations:** a Adult Restorative Dentistry, Oman Dental College, Muscat, Oman; b Associate Member of Staff & Honorary Researcher, Dundee Dental Hospital & School, Dundee, UK

**Keywords:** Cascade analysis, dentistry, endodontics, error, outcome

## Abstract

The ongoing endeavors to uncover the link between the prevalent errors in clinical endodontic training and undergraduate education are founded on tentative assumptions. This investigation was aimed at determining if cascade analysis can provide an understanding of the origins and causes of errors and if the sensitivity of student reports to the impact of errors on treatment outcomes can be established.In 2021, a group of investigators from the endodontics department concerned with clinical dental education launched the Study of Endodontic Errors (SEE). Sixty-six undergraduate dental students at one dental teaching hospital submitted anonymous narratives of problems they witnessed in their root canal treatment practices. The reports were examined to determine the sequence of events and the major errors. We kept track of the consequences of treatment outcomes, both as reported by students and as deduced by investigators.In 77% of the narratives, a chain of errors was recorded. The majority of the errors that took place were related to the working length or width of root canals. A substantial portion, 86%, of these errors could have been prevented through a deeper comprehension of the concepts that underlie working length and width. 75% of the errors that initiated cascades involved losing the correct working length. When asked whether the treatment outcome was compromised, students answered affirmatively in 16% of cases in which their narratives described compromised outcomes. Unacceptable outcomes necessitating re-treatment accounted for only 3% of student-reported consequences, but when investigator-inferred consequences were considered, the percentage more than doubled (7%).Cascade analysis of student error narratives is useful in understanding the triggering chain of events, but students provide insufficient information about how treatment outcomes are affected. Misconceptions about working length and width appear to play a significant role in the propagation of procedural errors.

## Introduction

The success of a root canal treatment procedure is dependent on various factors. From the student’s perspective, any hindrance to the smooth flow of the procedure can have a profound impact. In the dental field, such hindrances can be classified as either procedural errors or dental mishaps. Procedural errors refer to deviations from established protocols or procedures, while dental mishaps are unintended accidents. Understanding the consequences and identifying the factors that may have contributed to the occurrence of both procedural errors and dental mishaps is crucial, as they can have significant impacts on the treatment outcome.

Root canal treatment is a complex procedure that requires precision and expertise to ensure success. Despite its importance, studies exploring root canal treatment errors have been limited and have failed to provide a clear framework for measurement and definition. This study aims to fill that gap by delving into the intricacies of root canal treatment errors in an undergraduate teaching hospital and to shed light on all aspects that might have hindered the smooth flow of root canal treatment procedures from the student’s perspective, including both procedural errors and dental mishaps.

Most efforts to list procedural errors and mishaps during root canal treatment are aimed at providing insights and information about the associated issues and challenges, as well as educating practitioners on how to avoid them and improve the quality of treatment [1]. These efforts generally focus on downstream errors, particularly those related to instrument separation, ledge formation, and suboptimal obturation [1–4]. When viewed in isolation from their contributory origins, such errors can appear as clinical incompetence and trigger interventions focused on skill acquisition or calls for further research and development of the current set of tools and instruments [5,6]. However, the underlying issue may not always be directly influenced by the operator’s incompetence or the suboptimal properties of the instruments, but by the knowledge and understanding of concepts that were likely overlooked prior to the error. The negative consequences of overlooking errors that are precursory in nature can be significant and have been well documented across many medical disciplines [7,8]. Dentists operating with inadequate knowledge and understanding, along with the misperceptions this creates, may not be aware of avoidable errors that can occur early in treatment and pose the greatest threat to treatment outcomes.

Descriptions of dental ‘errors’ are crucial to comprehend the kind of errors taking place in dental practice and to create plans to enhance patient safety [9]. In dental clinics, incident reporting systems are established to collect information about events that have or may affect patients, including dental mishaps, instrument failures, equipment malfunctions, and close calls [9,10]. These reports play a vital role in improving the quality and safety of dental care. Despite the widespread implementation of these systems in many dental setups [11,12], there is a scarcity of comprehensive analysis and deep understanding of incidents within a single dental domain, particularly for procedures such as root canal treatment.

The conventional approach to dental incident reporting focuses solely on ensuring patient safety [13]. In this study, however, we take a different approach and examine difficulties or impediments that may have hindered the seamless execution of root canal treatment and, as a result, may have impacted the treatment outcome. While this approach may not be routine in everyday practice, we believe that it is essential to shed light on the potential cascades of errors that may occur during root canal treatment and to delve deeper into identifying the underlying causes or errors that may have contributed to the procedure unnoticed. By doing so, we aim to improve the execution and outcome of root canal treatment and enhance patient care.

The purpose of this study is to systematically categorize and analyze the errors made by undergraduate dental students during root canal procedures. The purpose of this analysis is to gain a more comprehensive understanding of the nature and prevalence of these errors. This information will be utilized to inform the design of targeted educational interventions aimed at decreasing the frequency of such errors in clinical practice.

## Methods

The Study of Endodontic Errors (SEE) was initiated in 2021 by a team of investigators from the endodontics department with a focus on clinical dental education. Prior to its commencement, the study received approval from the Institutional Review Board (IRB) at the hosting institution, validating its conformity with ethical guidelines. To ensure student anonymity and confidentiality of the submitted narratives, stringent measures were put into place.

From September 2021 to December 2022, 66 students who were in their fourth year at the start of the study and halfway into their fifth year by the study’s conclusion, at a dental teaching hospital were invited to participate. All consented and submitted anonymous narratives of incidents witnessed in their root canal treatment practices. This process was accomplished via a dedicated computer terminal set up to receive the submissions.

The students were instructed to submit narratives after each root canal treatment they completed, irrespective of whether they perceived any errors in the process. Thus, our dataset spans a spectrum of experiences, giving us a comprehensive view of the procedures within the endodontic training. Nevertheless, for the purposes of this study, we analyzed only those narratives containing errors or problems (Table 1). The narratives without identified errors have been collected for a separate investigation, the focus of which falls outside the scope of the current study.

**Table 1.** Questionnaire used in SEE 2021. The questionnaire used in this study was adapted and modified from the PCISME questionnaire (35) to better fit the specific needs and context of root canal treatment.

Study of Endodontic Errors (SEE) Questionnaire		
1	*Patient Demographic Information (Age and Gender)*	Free text
2	*Does the patient have a complex health issue?*	Yes/No
3	*Does the patient have a chronic health issue?*	Yes/No
4	*Incident Description: Please describe the incident, including its stage of occurrence.*	Free text
5	*Outcome: Please describe the actual and potential consequences of the incident.*	Free text
6	*Contributing Factors: Please identify any special circumstances that may have contributed to the incident.*	Free text
7	*Prevention Measures: Please suggest changes that could prevent a recurrence of the incident.*	Free text
8	*Quality of Treatment Outcome: Was the treatment outcome compromised by the incident?*	Free text
9	*Final Outcome Assessment: If the outcome was unacceptable, please rate its quality.*	5-point Likert scale
10	*Referral: Would you refer the patient to an endodontist for re-treatment or to a surgeon for extraction?*	Yes/No; Yes/No
11	*Incident Frequency: How often does this type of incident occur when treating your patients?*	5-point Likert scale
12	*Additional Comments: Do you have any other comments or observations regarding the incident?*	Free text

The narratives spanned both individual treatment sessions and completed treatments, with the students being explicit about the timeframe in their reports. Each report contained information about the tooth type being treated to provide context for the incidents.

The process of narrative analysis involved the development of a taxonomy for categorizing reported errors, including the identification and definition of key elements within the narratives. This methodological approach was informed by the seminal works of Mold (1986), Dovey et al. (2001), and Woolf et al. (2004), who underscored the importance of identifying the causal origins of errors, rather than focusing solely on immediate, downstream errors [14–16]. Their concepts surrounding cascade analysis and the ‘Toxic Cascade’ model were instrumental in shaping the decision to use student narratives as a critical source of data. This approach facilitated the tracing of error progression, from its genesis to its effects on treatment outcomes, within the unique context of dental clinical training. Furthermore, the methodology was guided by the broader, non-linear frameworks suggested by Godfrey-Faussett (2016) for understanding and improving clinical practice [17].

In December 2022, the error reports ‘narratives’ submitted by students were examined in order to outline the sequence of events described in the reports. A taxonomy was developed in which three potential methods were considered in order to categorize the errors reported in these events, and agreement was reached on the following elements:

### Error, narrative, cascade: definitions

**Figure 1.**
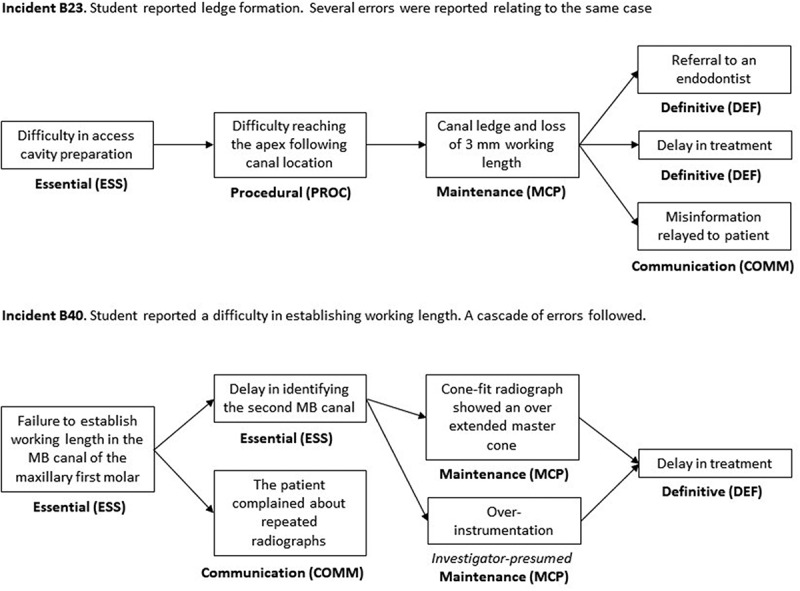
Examples of the cascade of errors identified in student-reported incident descriptions.

The errors within the incident narrative were defined as such, with the overall plot of what went wrong being defined as a narrative. A cascade was defined as a narrative involving multiple errors if one error led to another. Only actions or omissions that were inherently problematic or faulty were considered errors, regardless of any events that occurred before or after. If an error caused other events that were not errors, it was considered a single error rather than a cascade. Terminal errors, which were the final or ultimate errors in the cascade, such as not completing treatment or referring to an endodontic specialist or extraction, were defined as such. Precursor errors, which were the first or underlying errors in the cascades, were also defined. The chain of errors was graphically depicted to show the causal relationships (Figure 1). Only errors were listed, not all of the causal or predisposing factors that contributed to the incident. Contributing factors were only counted as errors if the group consensus was that they represented a problematic act. For example, a patient refusing to complete the treatment because it required multiple appointments was not coded as an error. Any errors that may have occurred during the incident but were not reported by the student, regardless of their likelihood, were not listed.

In our study, we have defined a series of interlinked errors leading to an ultimate undesired outcome as a ‘cascade of events’. We acknowledge that this terminology, though illustrative, may not be commonly used in existing literature. Drawing parallels from the studies by Haug et al. (2018) and Herbst et al. (2023), such a series of iatrogenic errors leading to a loss of working length can result in a suboptimal root filling length, affecting the overall quality of the treatment outcome [18,19]. This conceptual analogy lends credence to our usage of the term ‘cascade of events’ to describe a sequence of interrelated errors leading to a final outcome.

### Consequences

Consequences were defined as the effect on the final outcome of the treatment of errors. Although errors can occur and then be corrected, we only counted errors that affected the quality of the final treatment in this analysis. We divided the consequences into three categories [1]: injuries to the periapex (e.g., over extension of obturation) [2], errors that posed an increased risk of complications for the tooth after the reporting period, despite not having any immediate effect or those that could pose an increased risk but did not have consequences (e.g., presence of voids or short obturation), and [3] errors that led to referral to an endodontist for retreatment/management of errors or extraction. Whether the patient was inconvenienced by an unnecessary procedure, lost time, or other unexpected events was noted by the authors of this work when considering the latter, but no quantification was made.

Both [1] the consequences mentioned in the students’ narratives and [2] the consequences inferred by the investigators based on the narrative descriptions were taken note of. For example, it was deduced by the investigators that a short cone-fit radiograph error requiring a patient to undergo repeated periapical radiography was caused by an error in determining the working length of the canal, even if the student was unaware of the error. The inferred consequences were categorized into two groups: investigator-observed and investigator-presumed, depending on the level of certainty about their occurrence.

### Domains of procedures

Each reported error was classified into one of five root canal treatment procedure domains [1]: Essential (ESS) – errors in access cavity design, working length and width determination [2]; Procedural (PROC) – errors in handling instruments, materials, irrigation solutions, and medications [3]; Maintenance of crucial parameters (MCP) – failure to obturate the root canal to full length or failure to keep the obturation materials within the confines of the root canal space [4]; Communication (COMM) – errors in handling diagnostic tools, instructions, and patient record data, as well as interpersonal communication errors among students, instructors, and patients, or [5] Definitive (DEF) – errors delaying the application of a definitive restoration.

### Data analysis

The authors examined each narrative and came to a consensus on several aspects of the reported incidents, including the number of errors, which parts of the narrative constituted errors, the causal relationships depicted graphically, the best fit of each error into one of the five domains of care, the consequences reported by students and those observed or assumed by the investigators, and which aspect of the treatment outcome was most impacted by each consequence. Before reviewing the data, each investigator independently categorized the errors and consequences. Any disparities in the categorization were discussed as a group and the final codes were agreed upon through mutual decision. The flow of events was visually represented for each narrative and received unanimous approval.

After the coding was completed, descriptive statistics were gathered to describe the distribution of errors across the five procedure domains. A cascade analysis was performed to investigate the types of ‘precursor’ errors that took place earlier in the process, their patterns and sequences, and the ‘terminal’ errors that resulted. Lastly, an exploration was conducted into the distribution of consequences reported by students and those deduced by investigators.

## Results

Nine hundred narratives were reported, among which 1810 component errors were listed. The average number of reported narratives per student was 13.2, with a standard deviation of 3.1 and a range of 1–20. Two hundred seventeen (12%) of the component errors were single errors that did not trigger a cascade (i.e., ephemeral), but the remaining 1593 component errors were part of a chain of at least two errors. Out of the 900 narratives, 693 (77%) involved a cascade, among which 333 (48%) described a chain of two errors, 208 (30%) described a chain of three errors, and 152 (22%) described a chain of four errors (Figure 1).

The 900 narratives involved 210 ephemeral errors, 640 precursor errors, and 960 terminal errors as some narratives involved two or three precursor or terminal errors. Of the 960 terminal errors, 269 (28%) were ESS errors, 250 (26%) were DEF errors, 202 (21%) were MCP errors, 163 (17%) were COMM errors, and 77 (8%) were PROC errors (Table 2).

**Table 2.** Distributions of precursor and terminal errors within cascades.

Error Domain	Count
**Precursor errors**	
Essential (ESS) errors	N
● Failure to establish working length ● Failure to determine working width ● Difficulty in access cavity preparation ● Difficulty locating the orifice of an identified canal ● Delay in identifying a canal ● Losing the reference point in between sessions ● Problems in rubber dam placement ● Perforation	109 91 24 54 15 39 8 2
*Total*	**342**
Procedural (PROC) errors	N
● Difficulty reaching the apex following canal location ● Inability to keep length *(Investigator-presumed x 39)* ● Instrument separation ● Inability to dry the canal ● Sodium hypochlorite accident	84 57 (39^†^) 7 4 1
*Total*	**153**
Maintenance of Crucial Parameters (MCP) errors	N
● Canal ledge ● Loss of X mm working length ● Difficulty inserting the master gutta percha cone during cone-fit confirmation stage ● Overextended gutta percha master cone during cone-fit stage ● Short gutta percha master cone during cone-fit stage ● Over-instrumentation *(Investigator-presumed x 19)* ● Under – extended obturation ● Over-extended obturation	10 28 26 21 33 23 (19^†^) 17 12
*Total*	**140**
Definitive (DEF) errors	N
● Referral to an endodontist (e.g., instrument separation, perforation etc.)	2
*Total*	**2**
Communication (COMM) errors	N
● Patient failed to attend several consecutive times ● Patient arrived late	2 1
*Total*	**3**
***Precursor errors total***	***Reported (640)*** ***Reported + Presumed (698)***
**Terminal errors**	
Essential (ESS) errors	N
● Failure to establish working length ● Difficulty in access cavity preparation ● Difficulty locating the orifice of an identified canal ● Delay in identifying a canal ● Losing the reference point in between sessions	42 63 75 53 36
*Total*	**269**
Procedural (PROC) errors	N
● Difficulty reaching the apex following canal location ● Faulty length determination ● Instrument separation	36 34 7
*Total*	**77**
Maintenance of Crucial Parameters (MCP) errors	N
● Canal ledge ● Loss of X mm working length ● Difficulty inserting the master gutta percha cone during cone-fit confirmation stage ● Overextended gutta percha master cone during cone-fit stage ● Short gutta percha master cone during cone-fit stage ● Over-instrumentation ● Under – extended obturation ● Over-extended obturation	31 17 18 25 20 29 47 35
*Total*	**202**
Definitive (DEF) errors	N
● Referral to an endodontist (e.g., instrument separation, perforation etc.) ● Delay in treatment ● Compromised treatment outcome *(Investigator-presumed x 29)* ● Unacceptable outcomes necessitating re-treatment *(Investigator-presumed x 10)*	13 198 31 (29^†^) 8 (10^†^)
*Total*	**250**
Communication (COMM) errors	N
● Misinformation relayed to patient ● Difficulty addressing patient concerns ● Patient abandoned treatment	2 59 102
*Total*	**163**
***Terminal errors total***	***Reported (960)*** ***Reported + Presumed (999)***

^†^Investigator-presumed error (*n* = 39).

**Figure 2.**
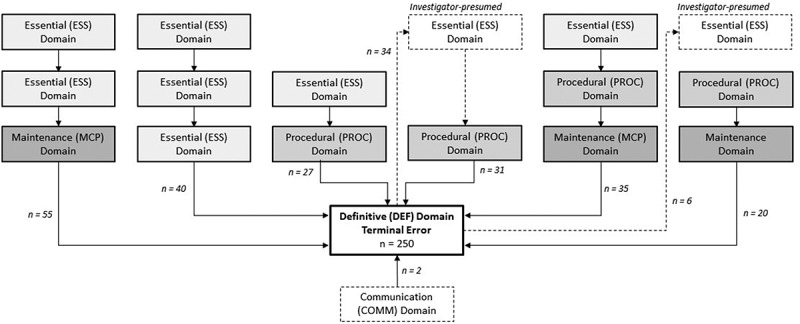
Precipitating errors of the 250 terminal errors in the definitive domain as depicted in the narratives.

217 narratives that did not involve a sequence of errors (ephemeral errors) were set aside, leaving 693 cascades for analysis. The precursor errors that precipitated the cascades were then examined. For the 250 terminal errors categorized under ‘DEF’, 143 (57%) were preceded by MCP errors. In 179 cases (71%) DEF errors were preceded by ESS errors, and in 40% of these cases 2 or more ESS errors precipitated the DEF error. It was also noticed that any error in essential steps always resulted in another ESS error or a PROC error before leading to an MCP or a DEF error via any cascade that ended in a terminal error (Figure 2).

**Figure 3.**
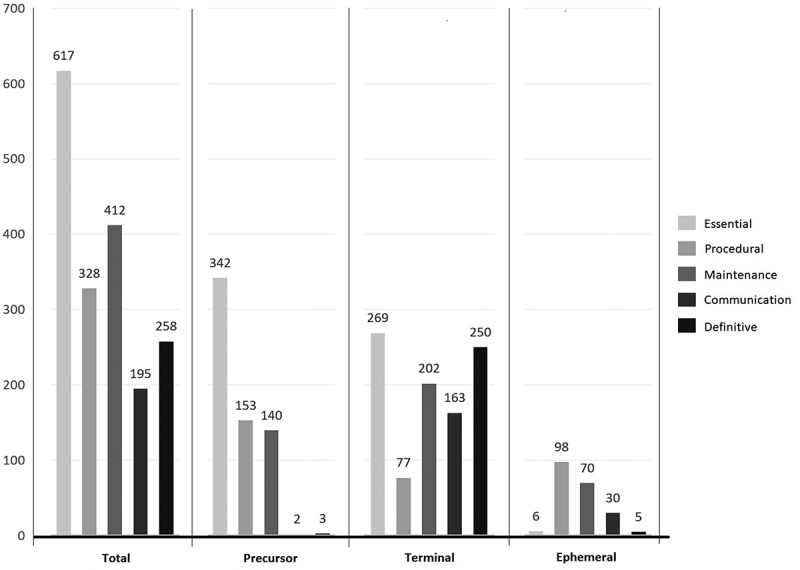
Error distribution across the five domains of root canal treatment procedure (*N* = 1810) reported in 900 narratives and for errors at both ends of the cascades, precursor and terminal (*N* = 640 and *N* = 960, respectively), and those that were not part of any cascade, ephemeral (*N* = 210).

One noteworthy observation was the relatively frequent occurrence of terminal errors stemming from errors in the ESS domain. Although errors in the PROC and MCP domains together accounted for only 29% of terminal errors, they accounted for the majority (80%) of ephemeral errors that did not generate a cascade (Figure 3). 87.5% of the errors that happened eventually came from errors in either the length or width of the work. A comparable pattern with the predominating ESS errors was observed at either end of the cascades, but not in the ephemeral domain where ESS errors were reported to be the least type of error. Several ESS errors often compounded or merged with one another, leading to terminal MCP or DEF errors. In total, ESS errors related to the working length and access cavity accounted for 75% and 37% of the narratives reported by students respectively.

Out of 480 working length-related precursor errors reported by students, 38% (182 errors) concerned difficulties in determining a corrected working length, 27% (130 errors) arose from losing the reference point during multiple appointments, 21% (101 errors) involved issues with confirming cone fit, and 14% (67 errors) manifested as obturation that was under- or over-extended. These first three error categories are potentially avoidable with a thorough understanding of root canal working length and width.

Out of the reported errors, not all could be attributed to problems with working length. 127 narratives were found to have originated from mistakes in the access cavity preparation, and 10 were caused by misdiagnosis. While these errors in clinical decision-making and root canal procedure may have also arisen from factors other than those mentioned, the students’ narratives did not provide evidence of such effects.

## Consequences to the treatment outcome

**Figure 4.**
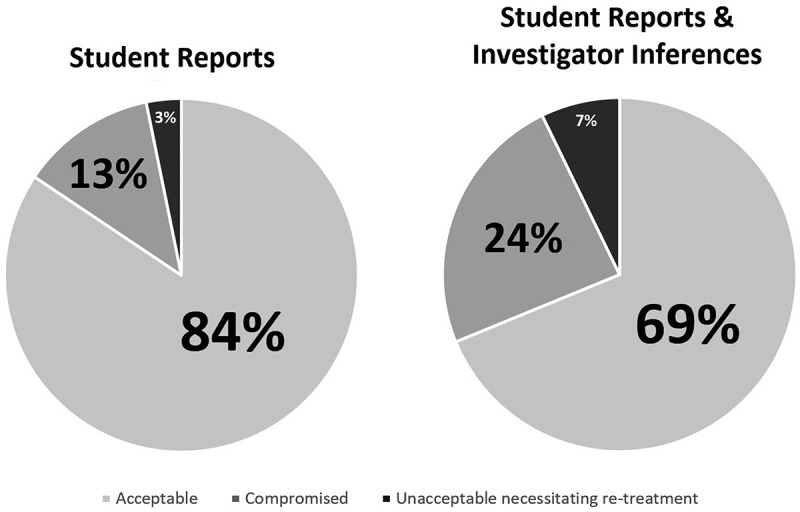
Comparison of treatment outcome consequences as self-reported by students only (left) and as combined with investigator inferences (right).

In thirty-nine (16%) of the student narratives, the students described a compromised treatment outcome as a consequence. However, when asked directly whether the outcome necessitates re-treatment, only 8 students (3%), answered affirmatively. Investigator analysis of the student reports revealed 29 additional observations in which the treatment outcome was likely affected but was not mentioned by the students, as well as 10 narratives in which an unacceptable outcome necessitating re-treatment is likely, raising the overall compromised outcome to 24% and unacceptable outcomes to 7% (Figure 4). As a result, investigator-presumed precursor errors thought to be attributed to post-analysis added consequences were found to be directly related to working length issues in 100% of the narratives, with nearly half of the narratives related to over-instrumenting the root canal apically (Figure 4).

## Discussion

This study delves into the intricate complexities of root canal treatment errors in an undergraduate teaching hospital. Before commenting upon the findings, a discussion of the rationale behind this work is warranted. Root canal treatment errors are often described as failed processes or unfortunate events, but their root causes are rarely explored. While many studies have compiled lists of possible errors in root canal treatment [20], only a few have attempted to measure these errors, and a clear framework for this measurement is lacking [2], as is the definition of error itself [21]. The absence of studies that trace adverse events back to their precursor errors through cascade analysis underscores the need for a more comprehensive understanding of root canal treatment errors. The potential benefits of using cascade analysis to identify latent procedure errors in root canal treatment have yet to be established in either the dental or endodontic literature.

Our study found the use of cascade analysis to be beneficial in identifying precursor errors, which are distinct from terminal errors and require different remedies. More than three quarters of the narratives in this study involved a series of interconnected errors. Documenting these error chains has several advantages. Firstly, it enhances the precision of error analysis by focusing on errors instead of incidents. Conventional error analysis may be skewed due to its methodology of categorizing each incident under a single name and counting it just once, such as under- or over-obturation, even when it is linked to other errors, like an incorrect working length determination or a lost reference point between appointments.

Inaccurate length determination can result in insufficient instrumentation and underfilling of the root canal, leading to persistent pain, discomfort, and inflammation of the remaining pulp tissue and periodontal ligament. This also increases the risk of procedural mishaps such as formation of a ledge, perforation, or transportation of the apical foramen. These complications make further treatment or retreatment challenging and may result in the necrosis of remaining pulp tissue, apical percolation, perpetuation of the periapical lesion, and a higher likelihood of treatment failure [22].

Secondly, cascade analysis uncovers the sequence of errors. Merely listing the errors related to root canal treatment does not provide insight into the cause-and-effect relationships or differentiate between the final errors and those that have a more significant impact. By clarifying the sequence of events, cascade analysis shifts the focus (and accountability) from the terminal error to the underlying circumstances that led to the precursor errors. Our findings showed that the majority of terminal errors were associated with mistakes in working length or width. However, we also discovered that at least 80% of these errors were triggered by mistakes that could have been avoided with a better understanding of the principles behind working length and width. In many cases, Even the most experiences dentist can fall for the same mistake if presented with the same inaccurate information.

Preclinical training is an essential aspect of dental education and plays a crucial role in preparing students for the complexities of root canal treatment. While the acquisition of clinical skills is crucial, it is equally important to emphasize the understanding of the rationale behind the clinical steps. This emphasis on understanding the concepts that underlie root canal treatment can lead to more effective and efficient learning.

Generally, students tend to focus more on the outcome of an experience rather than the process that leads to the outcome [23]. Similarly in dental education, one of the main challenges faced by dental students during preclinical training is a tendency to focus solely on the task of performing root canal treatment and shaping of the root canal space, to the exclusion of other important parameters. While the procedure can seem exhaustive, it is essential to recognize the importance of mastering these additional parameters, as they can significantly impact the overall success of the treatment. One of the reasons why students tend to focus more on the main task of shaping the root canal space is that it is often considered to be the most challenging aspect of the procedure. However, it is crucial to understand that while the acquisition of clinical skills will eventually occur through practice, the most effective way to learn these skills is through deliberate practice [24,25]. This type of practice involves not only performing the task repeatedly but also having a deep understanding of the rationale behind each step.

Preclinical training in dental schools is a comprehensive process, encompassing a variety of teaching methods. In addition to the emphasis on the task itself, sophomore students are often taught through a combination of lectures, clinical notes, and clinical demonstrations in phantom head labs and on real patients. Central to this teaching approach is the use of step-by-step guidelines derived from reference textbooks or prominent experts in the field [22]. However, while these guidelines serve as a vital framework for learning, they should not constrain the students’ ability to critically appraise the procedure and understand the rationale for each step. This can result in students blindly following the guidelines without a clear understanding of the underlying principles and concepts. Without a thorough understanding of the rationale behind each step, students may be less able to adapt to real-world clinical situations and make informed decisions. This is particularly relevant in root canal treatment, where every case is unique and requires a tailored approach. By fostering an understanding of the concepts that underlie the procedure, students can develop the critical thinking skills necessary to make informed decisions and achieve the best possible outcomes for their patients. Preclinical training must strike a balance between teaching students the step-by-step guidelines and fostering an understanding of the concepts that underlie the procedure. This will enable students to be better equipped to adapt to real-world clinical situations and make informed decisions, leading to better outcomes for their patients. By doing so, students can develop a more comprehensive understanding of the procedure and be better equipped to perform root canal treatment with confidence and competence. This shift in focus from solely acquiring clinical skills to understanding the rationale behind the clinical steps will lead to more effective preclinical training and better preparation for real-world clinical situations.

Additionally, the significance of working width in root canal treatment is often overlooked, as evidenced by the limited amount of research published on the subject. To verify this, a search was conducted on the Scopus database (www.scopus.com) on 10 February 2023, using the query ‘TITLE (“working width”) AND (LIMIT-TO (SUBJAREA, “DENT”))’. The search results were disappointing, yielding a mere four documents [26–29], with only two of them offering a conceptual perspective on the topic [28,29].

The working width of the root canal system is not only more intricate than the working length but also more challenging to assess. This is due to the significant variations in the horizontal dimension that occur at each vertical level of the canal. Routine clinical radiographs may give clinicians an erroneous perspective, causing them to adopt an inadequate plan for shaping the root canal system. Sadly, this crucial aspect of root canal treatment has been overlooked and insufficiently researched, and has not received adequate attention in preclinical dental education. As a result, some dental students and recent graduates may still harbor the misconception that all root canals are cylindrical in shape, based on what they observe in radiographs. Even experienced dentists, including endodontists, may be misled by false claims made by the manufacturers of certain root canal shaping systems. For instance, some systems assert that obturation can be considered sufficient and complete after the canal has been prepared using a finisher file that has a tip diameter of 200 µm. This statement ignores the anatomical fact that the diameter of all apical constrictions in teeth exceeds that of the file [30,31], which would lead to untouched and unprepared apical areas. This serves as a prime example of reliance on prescriptive guidelines rather than the ability to critically evaluate the source of information and tailor the approach for each case based on its unique circumstances.

It is worthwhile to note the systematic review by Aminoshariae & Kulild (2015), which provides a thorough analysis of the master apical file size and its impact on healing outcomes [32]. Their work underscores the complexity of endodontic treatment and the necessity of understanding its various facets. However, it also emphasizes our assertion that a broader comprehension of the canal’s dimensions – the ‘working width’ - is crucial, given its implications on effective root canal treatment.

In light of these challenges surrounding the understanding of the root canal system, our exploration of the ‘working width’ concept aims to address its overlooked importance in endodontic treatment planning. As for the reasons behind this oversight, we believe it may be due to a combination of factors. These include the tendency to overly simplify canal anatomy for instructional purposes, a potential deficit in educational emphasis on this aspect, and perhaps an over-reliance on guidelines that may not sufficiently underscore the concept of ‘working width’. This underscores the need for our research in providing a more comprehensive understanding of these intricate aspects of endodontic anatomy.

The third advantage of using cascade analysis is its ability to identify underlying upstream causes and provide solutions to both these problems and the errors they contribute to. For example, the majority of errors reported in this study indicate that they can be remedied by refining the preclinical training process. Cascade analysis directs resources and efforts towards the root causes and goes beyond traditional root cause analysis by identifying intermediary errors in the causal chain. The problems linking both ends of the cascade are easier to rectify than root causes and can be uncovered through the use of cascade analysis. In this study, the authors became more convinced in the effectiveness of the ‘content-specific instruction’ method, which involves understanding the learners’ misconceptions and the procedures for overcoming difficulties during clinical skill acquisition and execution [33]. The authors believe that clinical instructors should not only possess the ability to perform and supervise root canal treatment but also have a thorough understanding of the steps leading up to the final outcome, in order to effectively support learners regardless of their level of competence. This aligns with the notion presented by Shulman (1986) “*Those who can, do; those who understand, teach* [34]”.

Cascade analysis is widely utilized in the medical and dental fields to monitor progress towards successful outcomes, with a focus on ensuring patient safety [12,35]. However, the application of cascade analysis to track errors in specific procedures outside of the patient safety domain has yet to be fully explored in the dental field. This presents a missed opportunity for improvement and underscores the significance of incorporating cascade analysis in evaluating errors, leading to an elevated standard of dental care.

The significance of root cause analysis in comprehending dental errors is well established [12]. Kimura *et al*’s study of incident reports from a teaching dental hospital (2021) emphasized the importance of analyzing and comprehending the unique features of dentistry-related incidents and implementing suitable measures and educating dental professionals accordingly [11]. Currently, there is limited comprehensive analysis of incidents in the field of dentistry, and the specific nature of these incidents remains to be thoroughly investigated.

The review published by Estrela et al. in 2017 categorized operative procedural errors into a single table, albeit comprehensive, but their methodology was limited to listing the most frequently occurring errors based on the order of their occurrence during the clinical procedure without tracing the underlying causes. They emphasized that each operational error, regardless of its individual severity, can potentially impact the treatment outcome and increase the risk of failure [1]. Other studies have documented the common operational errors during root canal treatments and the possible consequences, such as postoperative complications, patient complaints, and legal ramifications [2,3]. Alghamdi et al.‘s observational study (2021), for instance, identified root canal obturation errors, with under-obturation being the most frequent, but did not address the potential underlying causes of these mishaps [3]. Merging the work of these previous authors with our notion of cascades indicates a more comprehensive and dynamic cause-and-effect model for root canal treatment errors.

Our findings indicated that students’ reporting was more effective in providing accounts of narratives rather than documenting the impacts on patients. Students seemed reluctant to acknowledge that treatment outcomes were compromised, even when the compromised outcome was mentioned in their narratives. This reluctance may stem from fear of self-criticism or a manifestation of denial [36]. The students’ focus was largely on immediate effects on the procedure, rather than the overall treatment outcome and prognosis. This lack of enthusiasm for these issues can be attributed to their prioritization of achieving a passing grade, rather than taking a self-reflective approach. This dichotomy remains an ongoing challenge that almost all institutions have yet to overcome [37].

The results of our research have several significant implications for the field of dentistry. Firstly, it is crucial to exercise caution when making claims about the prevalence of errors in specific dental procedures. Secondly, it is recommended to shift the focus of epidemiological studies and educational initiatives towards a more analytical approach, using techniques such as cascade analysis to uncover the root causes of errors and potential solutions. Thirdly, our findings, as well as those of others [3], suggest that a significant proportion of treatment errors are related to length. This highlights the importance of instructing students on the conceptual basis of each step of a procedure and fostering critical thinking skills in clinical education. Rather than simply listing possible errors as separate entities, the instructional method should focus on imparting a deeper understanding of the underlying principles and processes involved. This approach is likely to lead to a better understanding of the relationship between the steps of a procedure and may help reduce the number of treatment errors related to length. Fourthly, rather than striving for a universal error-reporting system, it is more crucial to ensure that the system provides sufficient information for cascade analysis. Our findings also indicate that student reporting may not be a reliable source of data on errors as students tend to underreport the impact of errors on treatment outcomes.

The frequent occurrence of conceptual mistakes related to working length and width in this study contrasts with the widely held belief that errors that pose the greatest risk to treatment outcomes are primarily procedural, due to a lack of adherence to strict treatment protocols [38]. The discrepancy between this belief and the dental community’s focus on procedural and operative complications of errors may, to some extent, reflect the greater level of practical experience in dealing with errors in general practice compared to the teaching dental hospital setting from which our data were derived. However, the minimal disagreement observed between students and the investigators of this study, who themselves practice endodontics, may suggest a divergence in perspective. This divergence could potentially stem from differing emphases within clinical practice dentistry and academic dentistry [39].

This may highlight a decline in academic dentistry, where its role in providing research and ideas to the profession may gradually diminish if the profit-driven dental industry and market continue to shape the profession uninterrupted. Therefore, relying on just one perspective is insufficient, and it’s essential to integrate multiple perspectives to fully grasp the frequency and impact of errors on treatment outcomes.

The methods and outcomes emphasized the significance of not just counting error types, but examining the events leading up to the final error. The results revealed that various terminal errors share common preceding errors, pointing towards potential areas for improvement. The study highlights the crucial importance of determining and preserving proper working length and the need to enhance students’ knowledge through training and education. The study’s conclusions were drawn not from identifying specific errors, but by comprehensively reviewing the entire process and presenting relevant descriptions of the sequence of events. The authors advocate for the involvement of other dental professionals in future error reporting systems. Although the observed under-reporting of consequences, the students’ willingness to report incidents and their progress make implementing such systems easier than anticipated. These contributions broaden the understanding of errors and suggest further avenues for research and intervention.

The importance of determining and preserving proper working length is emphasized and the need for enhancing students’ knowledge through training and education is highlighted in this research. The findings, based on a comprehensive review of the entire root canal treatment process, not only examine the types of errors but also the events leading up to them. The results reveal that various terminal errors share common preceding errors, pointing to potential areas for improvement. The authors advocate for the involvement of other dental domains in future error reporting systems and emphasize the students’ willingness and ability to contribute to such systems. This research represents a step forward in the understanding of root canal treatment errors and provides valuable information to guide future research and intervention. The authors hope that it will spark renewed interest in the field and encourage further exploration of the complex and interrelated factors that lead to errors in root canal treatment, ultimately with the goal of improving the quality of care and reducing error frequency in this critical dental procedure.

When interpreting the results of this study, several limitations should be taken into account. Firstly, the student reports used in the study may not have been entirely complete, accurate, bias-free, or representative of all errors in root canal treatment. This means that students may have omitted relevant details or reported incorrect causes. While the study did not aim to achieve representativeness or attribute blame, but rather to illustrate the concept of cascades using the elements of the narratives reported by students. This method could be utilized in future studies with more detailed documentation of incidents to gain a deeper understanding of the cause-and-effect chain. The cascade analysis in this study only focused on errors that seemed to propagate cascades and did not include other unidentified errors or predisposing factors that may have led to the errors. This was a deliberate simplification, but it’s crucial to recognize that a full cascade description would include all elements of the cause-and-effect chain. This is particularly important when considering the potential consequences of root canal treatment errors for patients. Additionally, it’s important to note that the cascades were created based on narratives and not an independent theoretical framework. Finally, the use of investigator judgment was necessary to determine causal relationships and assess the potential consequences for patients. However, this subjective approach may introduce bias into the analysis and limit the generalizability of the results. Further research is needed to gain a more comprehensive understanding of the causal chain and the potential consequences of errors in this field.

## Conclusion

The use of cascade analysis on student error reports has proven to be a valuable tool in comprehending the chain of events that lead to errors. However, students often provide limited information regarding the impact of these errors on treatment outcomes. Our findings suggest that misunderstandings regarding working length and width are a significant contributor to the occurrence of errors. By identifying and documenting these misconceptions, we can better understand the root causes of these errors and work towards reducing their frequency. Furthermore, this information can be used to develop educational strategies to improve students’ understanding of these critical concepts and enhance the quality of care provided to patients.

Our study has laid the foundation for the integration of cascade analysis of errors in root canal treatment within a teaching dental hospital setting, which can serve as a basis for future studies to assess the prevalence of mistakes in general practice. This knowledge can then be utilized to develop continuing professional development programs or modify current undergraduate curricula with the goal of reducing errors in root canal treatment.

## Data Availability

The data that support the findings of this study are available from the corresponding author upon reasonable request.
